# Maternal pre-pregnancy weight and early life lower respiratory tract infections in a low-income urban minority birth cohort

**DOI:** 10.1038/s41598-021-88360-y

**Published:** 2021-05-07

**Authors:** Maria J. Gutierrez, Gustavo Nino, Xiumei Hong, Xiaobin Wang

**Affiliations:** 1 Division of Pediatric Allergy, Immunology and Rheumatology, Johns Hopkins University, 600 N. Wolfe Street CMSC 1102, Baltimore, MD USA; 2 Division of Pediatric Pulmonary and Sleep Medicine. Children’s National Medical Center, George Washington University, Washington, DC USA; 3 Center On the Early Life Origins of Disease, Department of Population, Family and Reproductive Health, Johns Hopkins Bloomberg School of Public Health, Baltimore, MD USA; 4 Division of General Pediatrics & Adolescent Medicine, Department of Pediatrics, Johns Hopkins University School of Medicine, Baltimore, MD USA

**Keywords:** Diseases, Medical research, Risk factors

## Abstract

The prevalence of maternal obesity has increased dramatically with adverse consequences on infant health. Prior studies have reported associations between maternal obesity and childhood wheeze, asthma as well as lower respiratory tract infections (LRTI). However, studies examining the association of obesity with early-life LRTIs in low-income urban minority populations are still lacking. This is a critical gap because both obesity and infant respiratory morbidity are more prevalent and severe in these communities. We examined mother‐child dyads from the Boston Birth Cohort (BBC) to define the longitudinal association of maternal pre‐pregnancy BMI and LRTI in infancy, defined as the presence of bronchiolitis, bronchitis, or pneumonia during the first year of life (< 12 months of age). A total of 2,790 mother‐child dyads were included in our analyses. Infants born to pre-pregnancy obese mothers (n = 688, 25%) had 1.43 increased odds (adjOR = 1.43, 95% CI 1.08–1.88, p = 0.012) of developing LRTI during the first year of life when compared with newborns born to normal-weight mothers after adjusting by relevant LRTI risk factors. Notably, infants born to overweight mothers (n = 808, 29%) followed a similar trend (adjOR = 1.31, 95% CI 1.00–1.72, p = 0.048). Our study demonstrated that maternal pre-pregnancy obesity is an independent risk factor for the development of LRTI during infancy in a low-income urban minority birth cohort.

## Introduction

The incidence of obesity has reached epidemic levels in the U.S., affecting about 40% of adults^1^. Even more alarming is the increasing prevalence of maternal obesity and the negative consequences on infant health^2^. Approximately 50% of U.S. infants are born to overweight or obese mothers^3^ and many studies have reported deleterious effects of maternal obesity on the offspring. Several studies have reported an increased risk of wheeze and asthma in children born to obese mothers^4^. Other studies have identified a link between maternal obesity and lower respiratory tract infections (LRTI) in young infants^5^. The association between maternal obesity and early-life LRTI has critical implications for public health because LRTI are the world’s leading cause of death in young children and the top cause of hospitalization of infants in the U.S^6,7^. Moreover, LRTI in early life confer a substantially higher risk for subsequent chronic respiratory diseases, including wheezing and asthma^8^. Thus, it is critically important to better understand the link between obesity and early respiratory morbidity during the first year of life to develop prenatal primary prevention strategies for millions of infants born to obese mothers worldwide.

Prior British and Australian birth cohorts have already reported associations between maternal obesity and higher rates of hospitalizations due to respiratory infections among young children^5,9^. However, studies examining the association of obesity with early-life LRTIs in low-income urban minority populations are still lacking. This is a critical gap because both obesity and infant respiratory morbidity are more prevalent and more severe in these communities^10–12^. Moreover, the etiology of maternal obesity and pediatric LRTI is affected by socio-economic factors unique to low-income urban minority populations^12,13^. Also, prenatal and postnatal nutritional, environmental, and lifestyle factors shared within families and inner-city communities may affect the relationship between maternal obesity and LRTI risk. For instance, maternal smoking, prematurity, and ethnicity are strong risk factors for many respiratory disorders during early childhood, including LRTI, wheezing, and subsequent development of asthma^12,14,15^.

The goal of this study was to examine the relationship between maternal pre‐pregnancy obesity and LRTI during the first year of postnatal life in a prospective low-income urban minority birth cohort. The longitudinal relationship between maternal obesity pre-pregnancy obesity and LRTI during the first year of life was examined adjusting by relevant factors including maternal age, race/ethnicity, parity, history of smoking during pregnancy and infant’s delivery type, gestational age, sex, and breastfeeding status knowingly associated with increased risk of respiratory disease^14–17^.

## Methods

This study was based on data from the Boston Birth Cohort (BBC), which was established in 1998 and is ongoing to date^18^. Mother-infant dyads in the BBC are enrolled under protocols approved by the Institutional Review Boards (IRB) at Boston University Medical Center and the Johns Hopkins Bloomberg School of Public Health, and written informed consent is obtained from the mothers. All methods were carried out in accordance with relevant guidelines and regulations for involving human participants.

### Study population

**Figure 1:**
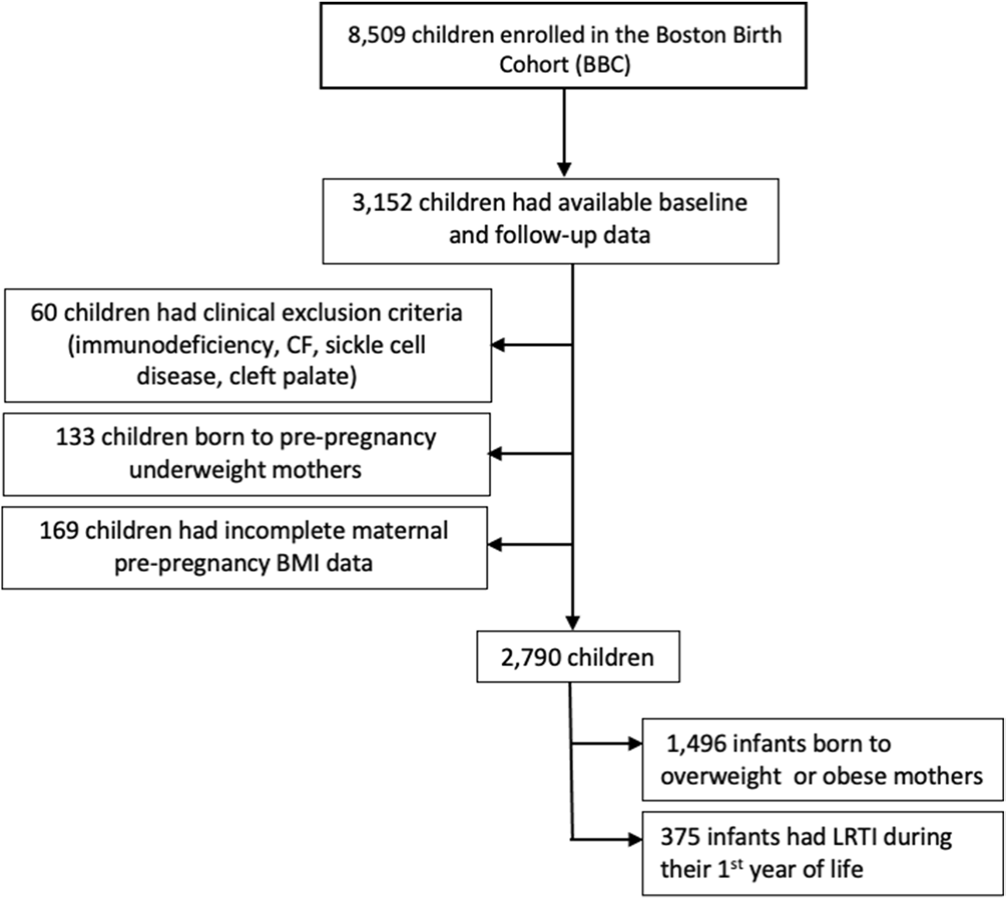
Study Population and outcomes.

Children in the BBC include singleton, live, premature, and full-term newborns recruited at the Boston Medical Center (BMC), which serves a predominantly low-income, minority, inner-city population in Boston, MA. Infants and mothers in the BBC are recruited at birth, and postnatal follow-up is conducted from birth to 21 years. Out of 8509 mother-infant pairs enrolled in the BBC between October of 1998 and March of 2019, 3,152 infants who had available postnatal follow-up records were included in the present study (Fig. 1). Their baseline characteristics are summarized and contrasted with those of excluded children in table S1. The prevalence of maternal pre-pregnancy overweight and obesity in relation to basic demographic factors of mothers enrolled in the BBC is also described in table S2. The BBC database contains all medical diagnoses recorded in electronic medical records for participants during the follow-up period. Clinical exclusion criteria for this study included a history of immunodeficiency, cystic fibrosis (CF), sickle cell disease, or cleft palate as defined by ICD-9 or ICD-10 diagnoses. Children born to pre-pregnancy underweight mothers, defined as body mass index (BMI) under 18.5, were omitted in order to include only mothers with pre-pregnancy normal weight in the comparison group and to avoid other forms of maternal malnutrition that could affect our results^19^. Finally, children with unavailable maternal pre-pregnancy BMI data were also excluded (Fig. 1).

#### Lower respiratory tract infections

The main outcome, lower respiratory tract infections (LRTI) in infancy, was defined as the presence of bronchiolitis, bronchitis, or pneumonia during the first year of life (< 12 months of age) and was identified by ICD9 or ICD10 diagnoses as recorded in the BBC database.

#### Maternal pre-pregnancy body mass index

As main predictor, we examined the effect of the pre-pregnancy maternal BMI, calculated from the mother’s self-reported height and weight before the current pregnancy. For this study, pre-pregnancy BMI was divided into categories, including mothers with normal pre-pregnancy BMI (BMI 18.5–24.9), overweight (BMI 25–29.9), or obese (BMI ≥ 30 kg/m^2^) before conception.

#### Definition of covariables

Additional predictors of LRTI were selected based on previous studies^14–17^ of determinants of childhood respiratory morbidity and included maternal factors such as maternal age at delivery, race/ethnicity, parity (first child or more), and maternal smoking during pregnancy. We additionally examined maternal education level as a social determinant of health, other cardiometabolic conditions during pregnancy (chronic and gestational diabetes, hypertensive disorders of pregnancy), and infant factors such as gestational age at birth, season of birth, delivery type, and breastfeeding (ever breastfed vs. never breastfed) during the first year of life.

### Statistical methods

We investigated the effect of maternal pre-pregnancy obesity on the development of LRTI during infancy (0–12 months of age). Exploratory data analysis was performed to inspect variables, detect outliers, and compare baseline demographic and clinical characteristics between infants who developed LRTI and those who did not. Continuous variables were summarized using means, medians, standard deviations, ranges, and interquartile ranges and analyzed using parametric or non-parametric methods as indicated. Categorical variables were tabulated. The percent of infants with missing data is also presented. To avoid excluding children with missing data in relevant covariates, incomplete observations in delivery type (0.4%), breastfeeding status (10.7%,) and pregnancy smoking (0.7%) were imputed using sequential imputation with chained equations in the multiple imputation (mi) function of STATA 14. Original and imputed datasets were reviewed and had comparable distributions. Twenty different datasets were generated, and their combined inferences are presented. The overall effect of pre-pregnancy maternal obesity on the occurrence of LRTI in the first year of life was estimated using single and multiple logistic regression. The predicted probability of LRTI during the first year for the values of BMI from normal, overweight and obese mothers (pre-pregnancy BMI 18.5–73.5 kg/m^2^) was also calculated. Model fitness was evaluated using the Akaike information criterion (AIC), and relevant confounders including maternal age, infant’s sex, prematurity, type of delivery, breastfeeding status were included. Factors knowingly important for the development of LRTI, including maternal race/ethnicity and history of maternal smoking, were locked into the model. Potential interactions between maternal BMI and breastfeeding, type of delivery and preterm birth on the odds of LRTI were also tested. Data were analyzed using the software R studio (RStudio: Integrated Development for R. RStudio, PBC, Boston, MA, 2020) and STATA version 14 (StataCorp. *Stata Statistical Software: Release 14*. College Station, TX, 2015).

## Results

A total of 3,152 children with available follow-up data after birth were screened for eligibility. There were 60 children with a history of immunodeficiency (n = 16), CF (n = 1), SCD (n = 39) and cleft palate (n = 4) who were not included (Fig. 1). Another 133 infants born to pre-pregnancy underweight mothers (pre-pregnancy BMI < 18.5) and 169 with incomplete BMI data were also excluded. A total of 2,790 infants were included in the analyses (Fig. 1) and their main demographic and clinical characteristics are summarized in Table 1.

**Table 1:** Demographic and clinical characteristics. Infants with LRTI were defined as those who developed bronchiolitis, bronchitis, or pneumonia during the first year of life as defined by ICD9 or ICD10 diagnosis in the Boston Birth Cohort (BBC) database. Maternal age, body mass index (BMI), and infant gestational age (GA) at birth are displayed as median and IQR (interquartile range) as their distributions were not Gaussian. There were significant differences in sex, parity, number of preterm infants, type of delivery, maternal smoking, and breastfeeding status between the two groups. There were also significant differences in maternal BMI and infant gestational age at birth between the group of infants who developed LRTI during the first year of life compared with those that did not. There were none or minimal amounts of missing data (< 1%) in most covariates, except for breastfeeding status that was unknown in ~ 11% of children.

Variable	All infants (n = 2,790)	Infants with LRTI (n = 375)	Infants without LRTI (n = 2,415)	p-value
**Sex (n, %)**				0.003
Female	1,377 (49.4)	158 (42.1)	1,219 (50.5)	
Male	1,413 (50.7)	217 (57.9)	1,196 (49.5)	
**Maternal race (n, %)**				0.234
White	205 (7.4)	27 (7.2)	178 (7.4)	
Black	1644 (58.9)	206 (54.9)	1438 (59.5)	
Hispanic	619 (22.2)	77 (23.2)	532 (22.0)	
Asian/Pacific Islander	44 (1.6)	6 (1.6)	38 (1.6)	
Other/mixed race	278 (10.0)	49 (13.1)	229 (9.5)	
**Maternal age in years**				0.797
Median (IQR)	28.3 (23.5–33.4)	28.2 (22.6–33.8)	28.3 (23.6–33.4)	
Maternal Education* (n, %)				0.710
No school/Elementary	116 (4.1)	16 (4.3)	100 (4.1)	
Some secondary school	622 (22.7)	94 (27.1)	528 (21.9)	
High school graduate/GED	1014 (36.3)	136 (36.3)	878 (36.4)	
Some college	626 (22.4)	79 (21.1)	547 (22.7)	
College degree and above	395 (14.2)	47 (12.5)	348 (14.4)	
Unknown	17 (0.6)	3 (0.8)	14 (0.6)	
Multiparous mother (n, %)	1626 (58.3)	242 (64.5)	1384 (57.3)	0.008
**Body Mass Index (BMI)**				0.006
Median (IQR)	25.6 (22.4–30.0)	26.6 (22.9–31.2)	25.5 (22.3–29.8)	
**BMI category (n, %)**				0.011
Normal	1294 (46.4)	148 (39.5)	1146 (47.5)	
Overweight (BMI 25–29.9)	808 (29.0)	117 (31.2)	691 (28.6)	
Obese (BMI ≥ 30)	688 (24.7)	110 (29.3)	578 (23.9)	
Gestational Age (weeks) (n, %)	38.7 (36.6–40.0)	38.1 (35.0–39.4)	38.9 (36.7–40.1)	0.000
Prematurity (< 37 wks.) (n,%)	789 (28.3)	141 (37.6)	648 (26.8)	0.000
Type of Delivery*				0.002
Vaginal Delivery	1,761 (63.1)	210 (56.0)	1551 (64.2)	
C-section	1017 (36.5)	165 (44.0)	852 (35.3)	
Unknown	12 (0.4)	0	12 (0.5)	
**Maternal diabetes (n,%)**	352(12.6)	294(12.2)	58(15.5)	0.074
Gestational Diabetes	222 (8.0)	34 (9.1)	188 (7.8)	
Type 1 or 2 Diabetes	130 (4.7)	24 (6.4)	106 (4.4)	
Hypertensive disorders of pregnancy* (n, %)	430 (15.4)	352 (14.6)	78 (20.8)	0.003
Preeclampsia, Eclampsia, HELLP syndrome	241 (8.6)	45 (12.0)	196 (8.1)	
Chronic Hypertension	189 (6.8)	33 (8.8)	156 (6.5)	
Unknown status	20 (0.7)	0	20 (0.8)	
Pregnancy smoking* (n, %)				0.048
Continuous smoking	307 (11.0)	54 (14.4)	253 (10.5)	
Unknown status	19 (0.7)	1 (0.3)	18 (0.8)	
Breastfeeding status* (n, %)				0.010
Breastfed infant (exclusive/partial)	1831 (65.6)	232 (61.9)	1599 (66.2)	
Unknown status	298 (10.7)	32 (8.5)	266 (11.1)	

IQR = Interquartile range, GED = Graduate Equivalence Degree, HELLP = hemolysis, elevated liver enzymes, low platelet count.

* Variables with missing data.

### Description of demographic and clinical characteristics

As previously described, the BBC is a large, predominantly urban, low-income minority birth cohort^18^. The comparison of the 3,152 infant-mother pairs who continued to follow in the BBC with the 5,357 without postnatal follow-up information, revealed differences in race, level of maternal education, type of delivery and proportion of obese mothers, prematurity and maternal smoking between the two groups and reflect the baseline characteristics of the population in our study (Table S1). Among the 3,152 mothers included, black women of African American or Haitian descent were the predominant racial group (58.9%), followed by Hispanics (22.2%), Whites (7.4%), Asian or Pacific Islanders (1.6%), and mothers from mixed or another races (10.0%). Among infants in this study, there was approximately the same number of males and females (50.7% and 49.3%, respectively), but a significantly higher number of males developed LRTI in the first year of life (57.9% vs. 42.1%, p = 0.003). The maternal level of education was heterogeneous, including one-fourth of mothers who had not completed high school, 36.3% who had graduated from high school and 36.6% who had attended or graduated from college. Most mothers included had had one or more children (58.3%), and there was a higher proportion of multiparous mothers with infants who developed LRTI compared with those who did not (64.5% vs. 57.3%, p = 0.008). In agreement with the existing literature, prematurity was associated with LRTI in infancy as 37.6% of children who developed LRTI in the first year were born before 37 weeks of gestational age compared with 26.8% in the non-LRTI group (p = 0.004). There were significant differences in delivery type (64.2% of infants were delivered vaginally in the non-LRTI group compared 56% with the LRTI group, p = 0.002), and there was a higher proportion of breastfed infants in the non-LRTI group (66.2%) compared with the LRTI group (56%) (p = 0.010). There were more mothers with preeclampsia/eclampsia or chronic hypertension in the LRTI group (20.8% vs. 14.6%, p = 0.003). Also, the proportion of mothers who smoked during pregnancy was higher in the group of infants with LRTI, compared with children without LRTI (14.4% vs. 10.5%, p = 0.048). Importantly, over one-half of mothers included in the final analysis started the current pregnancy overweight (29%) or obese (24.7%) and the median pre-pregnancy BMI was significantly higher among mothers of infants who had LRTI during infancy (p = 0.006). Conversely, there were no significant differences in race/ethnicity, maternal education level, maternal age, or maternal diabetes between the group of infants with LRTI and those without.

### Longitudinal association of maternal obesity and lower respiratory tract infection in early life

**Figure 2:**
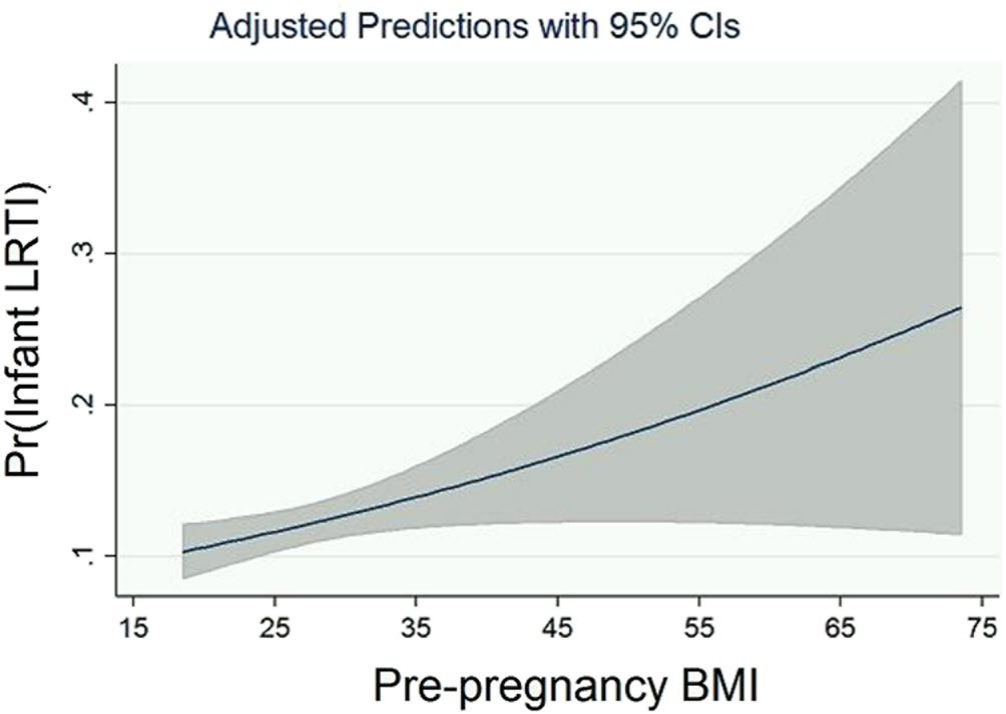
The predicted probability of LRTI during infancy increases with maternal pre-pregnancy BMI. Margins plot showing the increase in the predicted probability of LRTI during infancy (Pr(Infant LRTI)) from 0.115 (95% CI 0.10–0.13) to 0.288 (95% CI 0.14–0.44) as maternal pre-pregnancy BMI increased from 18.5 to 73.5 in the included infant-mother pairs.

A crude analysis of the relationship between maternal pre-pregnancy BMI and LRTI during the first year of life in the 2,790 infants included showed that maternal obesity was associated with 1.47 higher odds of LRTI in the offspring (OR = 1.47, 95% CI = 1.13–1.92, p = 0.004). The unadjusted odds of LRTI during infancy were also increased among 808 children born to overweight mothers compared to 1,294 children born to mothers with normal weight (OR = 1.31, 95%CI = 1.01–1.70, p = 0.041). The predicted probability of LRTI in the first year of life increased from 0.115 to 0.288 as maternal pre-pregnancy BMI increased from 18.5 to 73.5 (Fig. 2).

Adjusted analysis showed that infants born to obese mothers had 1.43-fold-increased odds of developing LRTI in infancy compared with infants born to normal-weight mothers (adjOR 1.43 95% CI = 1.08 -1.88, p = 0.012). Children born to overweight mothers exhibited a similar trend (adjOR 1.31, 95% CI 1.00–1.72, p = 0.048). Notably, this relationship was independent of maternal age, race/ethnicity, parity, history of smoking during pregnancy and delivery type, and from infant gestational age, sex, and breastfeeding status, also linked to the risk of LRTI in early life (Table 2).

**Table 2:** Multivariate analysis of the effect of maternal pre-pregnancy overweight and obesity on the odds of lower respiratory tract infections in the first year of life. The analysis was adjusted by age, race/ethnicity, parity, pregnancy smoking, delivery type, gestational age, infant sex, and breastfeeding status. P-values in bold represent statistically significant associations.

Variable		Infant LRTI (n = 2,790)	
		OR (95% CI)	p-value
**Maternal**	Pre-pregnancy BMI		
	Normal	Ref	
	Overweight	1.31 (1.00–1.72)	0.048
	Obesity	1.43 (1.08–1.88)	0.012
	Age at delivery	0.98 (0.96–1.00)	0.035
	Race/ethnicity		
	White	Ref	
	Black	1.00 (0.63–1.59)	
	Hispanic	1.16 (0.71–1.91)	
	Asian/Pacific Islander	1.49 (0.56–3.97)	
	Mixed/another race	1.64 (0.96–2.81)	
	Multiparity	1.42 (1.10–1.83)	0.006
	Pregnancy smoking	1.29 (0.91–1.82)	
**Infant**	Vaginal delivery	0.76 (0.60–0.96)	0.019
	Preterm	1.58 (1.25–1.99)	0.000
	Male sex	1.39 (1.11–1.74)	0.004
	Breastfed infant	0.76 (0.57–0.99)	0.040

## Discussion

Our study demonstrated that maternal pre-pregnancy overweight and obesity are independent risk factors for the development of LRTI during infancy in a large birth cohort of predominantly minority, inner-city mothers, and infants. Our findings are highly relevant because over 50% of U.S. mothers are overweight or obese at the beginning of pregnancy^3^, minority women are disproportionally affected by the obesity epidemics^10^, and LRTI are the top cause of hospitalization among U.S. infants and a top cause of infant mortality worldwide.

Importantly, our findings are in agreement with previous studies in young children from other diverse backgrounds^5,9,20^. Notably, the link between maternal obesity and lower respiratory tract infections in infants under one year of age was demonstrated by Rajappan et al., who described a LRTI relative risk of 1.23 among children born to obese mothers in a longitudinal birth cohort of 2,799 British infant-mother pairs^5^. Associations between maternal obesity and hospitalizations due to respiratory infections are also described by Gutvirtz et al. in a population-based cohort of 249,840 Israeli children^20^ and by Cameron et al., who showed that during the first five years of life, children born to obese mothers had more hospitalizations for infectious diseases and respiratory conditions than children born to mothers with normal pre-pregnancy body mass index in a birth cohort of 2,807 Australian children^9^.

These findings suggest that early life events related to maternal pre-pregnancy obesity predispose to respiratory infections in infancy. We believe that multiple mechanisms could be mediating this relationship^21–23^. For instance, epigenetic changes (e.g., DNA methylation, histone modifications, and non-coding RNAs) are determinants of embryonic and fetal tissue and cell development and may be altered by maternal obesity^22,24,25^. As metabolism is a major regulator of immune cell function^26^ and knowingly associated with increased susceptibility to infection in other ages^27^, maternal obesity-related metabolic dysregulation could impair infant host defense and contribute to LRTI risk. Indeed, studies have shown that maternal obesity leads to metabolic derangements (e.g., fatty acids metabolism)^28^ and impacts fetal immune system maturation and infection susceptibility^21,29,30^. Clinically, data from the COPSAC (Copenhagen Prospective Study of Asthma in Childhood) birth cohort revealed that maternal supplementation with n-3 omega polyunsaturated fatty acids (PUFA) during healthy pregnancies decreased the incidence of early life LRTI in the offspring supporting a direct link between maternal metabolic status and LRTI risk^31^. At the cellular level, metabolism is considered a guiding force for immunity playing a central role during development, activation, and lineage commitment of immune cells^26^. For instance, glucose metabolism, aerobic glycolysis, oxidative metabolism, the citric acid cycle, and levels of amino acids (or of other metabolites) regulate differentiation and activation of macrophages, T cells, B cells, and NK cells^26^. Alternatively, changes in the infant microbiome have been demonstrated in animal models and human studies of maternal obesity^32,33^ and obesity-related changes in the microbiome, recognized as a regulator of early-life immune development^34^, could also mediate LRTI risk. There may be effects of maternal obesity on additional factors regulating immunity (e.g., maternal transfer of antibodies, immune factors, and cells to the newborn)^35,36^ that are not yet elucidated. Finally, as immune system development is highly influenced by the environment^37,38^, maternal and infant living conditions, as well as obesity-related environmental factors (e.g., diet, lifestyle), may be contributing to shape early life immune responses.

Our study has some limitations. First, because this is an observational study, we are not able to establish causal relationships. Second, although the definition of medical diagnoses using ICD9/10 codes allowed us to include a large number of children, the assessment of clinical details of LRTI and other clinical outcomes was not possible. Additionally, although self-reporting of pre-pregnancy weight and height allowed the ascertainment of BMI in a large number of mothers enrolled in the BBC (> 93%), recall errors may occur. Although we included most of the known determinants of LRTI risk, there are other potential confounders (e.g., infant’s weight gain, maternal history of asthma) that were not included in our model. Similarly, although we examined the relation between LRTI with maternal education and LRTI risk, the effect of other socio-economic indicators (e.g., household income) was not described and should be further studied. Of note, the frequency of LRTI among infants in our cohort was lower in comparison with previous data^7^. It is possible that by including only physician-diagnosed cases, we have captured only the most severe spectrum of LRTI that required medical attention, and asymptomatic or mild episodes have not been reported. In addition, the possibility that LRTI cases are not reported in our database (e.g., seen at other institutions outside BMC), although low, cannot be completely excluded.

Nevertheless, the identification of pre-pregnancy maternal obesity as an associated factor behind the development of LRTI during infancy in a predominantly urban, low-income, minority population is important. First, our findings are in agreement with existing studies demonstrating that pre-pregnancy maternal obesity is an independent predictor for the development of early life LRTI in children from multiple backgrounds^5,9,20^. Hence, it highlights a common preventable risk factor of infant respiratory morbidity. Considering that minority underserved women of childbearing age are disproportionally affected by obesity^10^, studies aimed to understand and prevent the effect of maternal obesity in infant respiratory health will be directly relevant to the most vulnerable segments of the U.S. population: urban, low-income, minority women, and their infants.

Importantly, our findings support the hypothesis that intrauterine life is important for the development of early life immunity and motivates further investigation into the mechanisms underlying LRTI risk in early life, which can eventually point to prevention^5,9,20,30,39^. The notion that infant immunity may be influenced by *in-utero* exposures also prompts to study whether other pediatric immunological diseases (e.g., allergies, autoimmunity) may have prenatal determinants. Furthermore, research aimed to better understand normal human immune development may provide clues about how rare and common immunological diseases emerge.

### Conclusion

The impact of this study is that it provides novel evidence of a strong association between maternal obesity and early-life LRTI in a low-income, urban, minority community independently of known LRTI risk factors. These results raise important questions about why maternal obesity increases the vulnerability to respiratory pathogens in early life. Elucidating the underlying disease mechanisms of how maternal obesity and early-life LRTI are linked is key to understand the origin of several respiratory conditions associated with increased maternal weight. Further investigating this link could also lead to novel strategies for risk stratification of infants at high-risk of LRTI and primary prevention in children born to obese mothers in urban, high-risk, underserved populations.

## Supplementary Information

Supplementary Information

## Data Availability

The datasets generated during and/or analyzed during the current study are available from the corresponding author on reasonable request after review and approval of the Institutional Review Board.
