# Supplementary material for: Maternal pre-pregnancy weight and early life lower respiratory tract infections in a low-income urban minority birth cohort
Source: Sci Rep. 2021 May 7;11:9790. doi: 10.1038/s41598-021-88360-y (PMC8105349; doi:10.1038/s41598-021-88360-y)
Supplement: Supplementary file 1 — Supplementary Information [file 41598_2021_88360_MOESM1_ESM.docx]

**MATERNAL PRE-PREGNANCY WEIGHT AND EARLY LIFE LOWER RESPIRATORY TRACT INFECTIONS IN A LOW-INCOME URBAN MINORITY BIRTH COHORT**

Maria J. Gutierrez,^a^ Gustavo Nino ^b^ , Xiumei Hong ^c^ and Xiaobin Wang^c,d^

1. Division of Pediatric Allergy, Immunology and Rheumatology, Johns Hopkins University, Baltimore, MD, USA.
2. Division of Pediatric Pulmonary and Sleep Medicine. Children’s National Medical Center, George Washington University, Washington, D.C., USA
3. Center on Childhood Origins of Disease; Department of Population, Family and Reproductive Health; Johns Hopkins Bloomberg School of Public Health; Baltimore, MD, USA.
4. Division of General Pediatrics & Adolescent Medicine, Department of Pediatrics, Johns Hopkins University School of Medicine, Baltimore, MD, USA

**SUPPLEMENTARY MATERIALS**

| *Variable* | *Infant-mother dyads excluded (n=5,357)* | *Infant-mother dyads included (n=3,152)* | *p-value* |
| --- | --- | --- | --- |
| *Child’s Sex (n, %)* |  |  | 0.382 |
| *Female* | 2,709 (50.6) | 1,563 (49.6) |  |
| *Male* | 2,648 (49.4) | 1,589 (50.4) |  |
|  |  |  |  |
| *Maternal Race (n, %)* |  |  | 0.000 |
| *White* | 776 (14.5) | 230 (7.3) |  |
| *Black* | 2184 (40.8) | 1,847 (58.6) |  |
| *Hispanic* | 1,721 (32.1) | 702 (22.3) |  |
| *Asian/Pacific Islander* | 139 (2.6) | 51 (1.6) |  |
| *Other/mixed race* | 537 (10.0) | 322 (10.2) |  |
|  |  |  |  |
| *Maternal Age in years* |  |  | 0.000 |
| *Median (IQR)* | 27.6 (22.8-32.7) | 28.2 (23.3-33.4) |  |
|  |  |  |  |
| *Maternal Education* (n, %)* |  |  | 0.000 |
| *No school/Elementary* | 393 (7.3) | 133 (4.2) |  |
| *Some secondary school* | 1,363 (25.4) | 741 (23.5) |  |
| *High school graduate/GED* | 1,652 (30.8) | 1,141 (36.2) |  |
| *Some college* | 956 (17.9) | 684 (21.7) |  |
| *College degree and above* | 855 (16.0) | 431 (13.7) |  |
| *Unknown* | 138 (2.6) | 22 (0.7) |  |
|  |  |  |  |
| *Multiparous mothers (n, %)* | 3,037 (56.7) | 1,809 (57.4) | 0.529 |
|  |  |  |  |
| *Maternal overweight or obesity* (n,%)* | 2,237 (41.8) | 1,526 (48.4) | 0.000 |
| *Unknown* | 407 (7.6) | 172 (5.5) |  |
|  |  |  |  |
| *Prematurity (<37 weeks GA)* (n, %)* | 1,406 (26.3) | 902 (28.6) | 0.018 |
| *Unknown* | 7 (0.13) | 4 (0.13) |  |
|  |  |  |  |
| *Type of Delivery* (n, %)* |  |  | 0.000 |
| *Vaginal* | 3,646 (68.1) | 2,012 (63.8) |  |
| *C-section* | 1,634 (30.5) | 1,127 (35.8) |  |
| *Unknown* | 76 (1.4) | 13 (0.4) |  |
|  |  |  |  |
| *Pregnancy smoking* (n, %)* |  |  | 0.003 |
| *Continuous smoking in pregnancy* | 691 (12.9) | 339(10.8) |  |
| *Unknown status* | 49 (0.9) | 19 (0.6) |  |

**Table S1. Comparison of the baseline characteristics of included and excluded infants and mothers.** Baseline basic demographic and clinical characteristics at birth were available in 8,509 infant-mother dyads. A total of 3,152 infants had follow-up data available and were included in this study. There were differences in race/ethnicity and maternal level of education, maternal age, type of delivery and maternal smoking between included and excluded infants. Additionally, there was a higher proportion of mothers with maternal pre-pregnancy overweight or obesity, and of babies delivered prematurely in the group that continued to follow-up.

| *Variable* | *All mothers*  *(n=8,509)* | *Non-obese mothers (n=4,167)* | *Overweight or Obese Mothers (n=3,763)* | *p-value* |
| --- | --- | --- | --- | --- |
| *Maternal race (n, %)* |  |  |  | **0.000** |
| *White* | 1,006 (11.8) | 601 (14.4) | 373 (9.9) |  |
| *Black* | 4,031 (47.4) | 1,738 (41.7) | 2,042 (54.3) |  |
| *Hispanic* | 2,423 (28.5) | 1,186 (28.5) | 994 (26.4) |  |
| *Asian/Pacific Islander* | 190 (2.2) | 152 (3.7) | 35 (0.9) |  |
| *Other/mixed race* | 859 (10.1) | 490 (11.8) | 319 (8.5) |  |
|  |  |  |  |  |
| *Maternal Marital Status (n, %)* |  |  |  | **0.007** |
| *Married* | 2,871 (33.7) | 1,346 (32.3) | 1,324 (35.2) |  |
| *Single* | 5,220 (61.4) | 2,631 (63.1) | 2,242 (59.6) |  |
| *Other* | 249 (2.9) | 113 (2.7) | 117 (3.1) |  |
| *Unknown* | 169 (2.0) | 77 (1.9) | 80 (2.1) |  |
|  |  |  |  |  |
| *Multiparous mother (n, %)* | 4,846 (56.9) | 2,089 (50.1) | 2,416 (64.2) | **0.000** |
|  |  |  |  |  |
| *Maternal age in years* |  |  |  | **0.000** |
| *Median (IQR)* | 27.8 (23.0-32.9) | 26.6 (22.1-31.7) | 28.9 (24.0-34.1) |  |
|  |  |  |  |  |
| *Maternal Education* (n, %)* |  |  |  | **0.000** |
| *No school/Elementary* | 526 (6.2) | 216 (5.2) | 216 (5.7) |  |
| *Some secondary school* | 2,104 (24.7) | 1,065 (25.6) | 859 (22.8) |  |
| *High school graduate/GED* | 2,793 (32.8) | 1,325 (31.8) | 1,298 (34.5) |  |
| *Some college* | 1,640 (19.3) | 793 (19.0) | 798 (21.2) |  |
| *College degree and above* | 1,286 (15.1) | 698 (16.8) | 520 (13.8) |  |
| *Unknown* | 160 (2.0) | 70 (1.7) | 72 (1.9) |  |

**Table S2.** **Demographic characteristics of participant mothers compared by body mass index (BMI) category.** There were 3,763 mothers (44.2%) affected by pre-pregnancy overweight or obesity, 4,167 non-obese mothers (48.9%) and pre-pregnancy BMI was unknown in 579 mothers (6.8%). There were statistically significant differences in several maternal characteristics including race, marital status, level of education, age at delivery and history of multiparity between obese and overweight mothers in comparison with non-obese mothers.
